# Zero leprosy is within reach: eliminating leprosy in low-endemic settings demands political will

**DOI:** 10.1016/j.lansea.2025.100703

**Published:** 2025-12-05

**Authors:** Anil Fastenau

**Affiliations:** a Department of Global Health, Institute of Public Health and Nursing Research, University of Bremen, Bremen, Germany; b Marie Adelaide Leprosy Centre (MALC), Karachi, Pakistan; c German Leprosy and Tuberculosis Relief Association (GLRA/DAHW), HQ, Wuerzburg, Germany

Pakistan's trajectory offers a critical proof of Zero Leprosy concept for South and Southeast Asia: the last mile of leprosy elimination is technically achievable, but it will not be reached through medical solutions alone. The country has reduced new leprosy case numbers from 971 in 2001 to just 236 in 2023, despite immense health system pressures, humanitarian crisis spillover, and shifting political priorities.^1^ According to national surveillance data, the number of new leprosy cases has declined steadily over the past 20 years, with adult cases declining by 75%, from 878 cases in 2001 to 220 cases in 2023.^1^ The number of child cases (younger than 15 years) has declined by 83%, from 93 cases in 2001 to 16 cases in 2023.^1^ This decline is not a passive epidemiological trend. It reflects deliberate, long-term investments in community engagement, data-driven targeted active case finding and sustained NGO-government collaboration. Pakistan now sits at a strategic tipping point, either to cross the final threshold of transmission interruption or to plateau like several low-endemic countries did before it.

Despite significant progress towards zero leprosy in Pakistan, ongoing transmission is still suggested by 16 new child cases reported in 2023 and a continued high proportion of multibacillary (MB) presentations.^1^^,^^2^ The presence of Grade 2 Disability among more than one-third of newly diagnosed patients in Karachi reflects persistent diagnostic delays, with a mean delay of three years and a median of two years among recently diagnosed cases (n = 150).^3^ These numbers highlight a structural truth: low-endemic does not mean low priority, in fact, it demands greater precision, targeted active detection and stronger health system integration. Three strategic elements explain Pakistan's achievements and offer transferable lessons for the region.

First, systematic retrospective contact tracing and post-exposure prophylaxis (PEP) have moved from pilot to pathway. Pakistan is the only South Asian country implementing retrospective contact tracing and examination up to 10 years back, enabling the identification of undiagnosed leprosy cases that conventional passive surveillance would never detect. When combined with SDR-PEP, these interventions show strong impact in reducing future case incidence.^4^^,^^5^ Country-wide scale-up requires firm political commitment, predictable financing, and full integration within national skin-NTD frameworks.

Public–private collaboration is the backbone of Pakistan's progress. NGOs such as Marie Adelaide Leprosy Centre (MALC) and Aid to Leprosy Patients (ALP) have maintained over five decades of uninterrupted field presence, preserving data continuity, community trust and operational speed independent of political cycles.^1^^,^^6^ This continuity, not externally driven funding cycles or short-term innovations, is the true value driver in the last mile. Many neighboring countries lack such infrastructure, making Pakistan's model worth strategic adaptation rather than replication.

Beyond community-led approaches, new digital tools now offer opportunities for more precise and proactive leprosy control. Pakistan has begun to deploy modern intelligence tools, but their full potential is still unrealized. Geographic Information System (GIS) mapping and digital surveillance platforms such as case-based DHIS2 and WHO's Leprosy Elimination Monitoring Tool (LEMT) now enable real-time hotspot analysis, shifting strategy from reactive to anticipatory.^7^ However, these systems remain underfinanced and disconnected from national surveillance pipelines. This is the moment to institutionalize them, before donor fatigue forces scaling down rather than scaling up.

Yet Pakistan's own experience also reveals the central blind spot in global leprosy elimination: mental health and stigma remain secondary in strategy and funding, despite direct links to diagnostic delay, treatment adherence and social reintegration.^8^ Human rights frameworks are referenced but rarely operationalized. Storytelling and peer-led advocacy initiatives show transformative potential, especially among women and young people affected by leprosy,^9^ yet they are still perceived as “soft components”, not strategic drivers of elimination outcomes.

The single greatest regional risk is political complacency. South Asia has entered the 2030 endgame with declining prevalence but not declining urgency. If integration within broader national skin NTD programs does not occur now, as successfully piloted in Togo, Pakistan faces the same stagnation currently seen in similarly low-endemic countries where transmission flatlines but does not disappear.^10^ Securing political will in low-endemic settings requires linking leprosy elimination to broader health priorities rather than competing with them. Practical strategies include embedding Zero Leprosy activities within national skin-NTD programs, demonstrating cost-effectiveness through routine data, and aligning interventions, such as contact tracing and PEP, with existing primary-care and universal health coverage (UHC) agendas. Strengthening evidence-based advocacy, engaging civil-society partners, and framing leprosy elimination as a governance success rather than a vertical demand can also help sustain high-level commitment despite competing priorities.

The lesson from Pakistan is not that the last mile is easy, but that it is feasible. And that possibility depends on four non-negotiables: (1) sustained political financing (2) institutionalized integration within national skin NTD frameworks (3) digital intelligence for proactive, rather than passive, surveillance (4) treating stigma and mental health as core determinants, not optional add-ons.

This is the moment for South and Southeast Asia to act decisively and translate political commitments into sustained, institutionalized action. Pakistan has shown the pathway: grounded in data, community trust and long-term partnership rather than pilot-driven innovation cycles. The only remaining question is whether political courage, not just technical capacity, will match that pathway. Elimination will depend not on new tools, but on ensuring that political commitment and system-wide integration keep pace with epidemiological progress.

## Contributors

AF: Conceptualisation, literature review, writing—original draft, coordination, writing—review & editing.

## Declaration of interests

The author declares no conflict of interest.
